# Efficacy of intravitreal bevacizumab combined with pan retinal photocoagulation versus panretinal photocoagulation alone in treatment of proliferative diabetic retinopathy

**DOI:** 10.12669/pjms.331.11497

**Published:** 2017

**Authors:** Murtaza Sameen, Muhammad Saim Khan, Ahsan Mukhtar, Muhammad Amer Yaqub, Mazhar Ishaq

**Affiliations:** 1 Dr. Murtaza Sameen, MBBS. Armed Forces Institute of Ophthalmology, Rawalpindi, Pakistan; 2 Dr. Muhammad Saim Khan, MBBS, FCPS, FICO, MRCSEd. Armed Forces Institute of Ophthalmology, Rawalpindi, Pakistan; 3 Dr. Ahsan Mukhtar, FCPS (Ophth), FCPS (Vitreoretina), FRCS (G). Armed Forces Institute of Ophthalmology, Rawalpindi, Pakistan; 4 Dr. Muhammad Amer Yaqub, MCPS, FCPS, FRCSEd. Armed Forces Institute of Ophthalmology, Rawalpindi, Pakistan; 5 Prof. Dr. Mazhar Ishaq, FCPS, FRCOphth, FRCSEd. Armed Forces Institute of Ophthalmology, Rawalpindi, Pakistan

**Keywords:** Diabetic macular edema, Laser pan-retinal photocoagulation, Intravitreal Bevacizumab, Proliferative diabetic retinopathy

## Abstract

**Objective:**

To compare effectiveness of pan-retinal photocoagulation alone versus panretinal photocoagulation combined with intravitreal bevacizumab on visual acuity and central macular thickness in patients presenting with proliferative diabetic retinopathy.

**Methods:**

This Randomized controlled trial was carried out at Armed Forces Institute of ophthalmology, Pakistan from Jan 2016 to Aug 2016. Seventy six eyes of 50 patients having proliferative diabetic retinopathy and diabetic macular edema were included in the study. All the patients were subjected to detailed clinical examination that included Uncorrected visual acuity (UCVA), best corrected visual acuity (BCVA), slit lamp examination of anterior and posterior segments. Optical coherence tomography (OCT) and fundus fluorescein angiography (FFA) were carried out and patients were divided in two groups (GP and GI). Three monthly sessions of Pan retinal photocoagulation (PRP) using Pattern Scan Laser (PASCAL) alone was performed in group GP while PRP along with three monthly intravitreal bevacizumab (IVB) was performed in group GI. BCVA and CMT was recorded 04 weeks after the third PRP session in both the groups.

**Results:**

Seventy six eyes of 50 patients (38 in each group) were treated with three sessions of PRP alone and PRP with IVB in Group GP and GI respectively. Mean age of the patient in group GP was 57.47± 6.08 years while that in group GI was 55.69 ±6.58. The magnitude of induced change in BCVA was 0.09 ± 0.15 in GP while 0.22 + 0.04 in GI groups while mean induced change in CMT after treatment was 77.44 ± 92.30 um and 117.50 ± 93.82 um in group GP and GI.

**Conclusion:**

Laser PRP combined with IVB has superior visual and anatomical outcome than PRP alone in patients with combined presentation of PDR and DME.

## INTRODUCTION

Diabetes Mellitus is a chronic, metabolic multisystem disorder affecting working age population all over the world and 11.7% of Pakistani population.^1^,^2^ Diabetic retinopathy (DR) is an important microvascular complication of diabetes affecting almost 36% of diabetic population. It is one of the commonest cause of visual loss.^3^ Diabetic macular edema (DME) and proliferative diabetic retinopathy (PDR) are two prime manifestations of DR that are responsible for visual morbidity. The mechanism involved in development of DME and PDR is raised VEGF levels secondary to ischemia and hypoxia induced by diabetic microvascular changes.^4^

The mainstay of treatment in PDR is Laser photocoagulation as concluded by Diabetic retinopathy treatment study (DRS) and Early treatment diabetic retinopathy study (ETDRS) for the last couple of decades.^5^ According to early treatment diabetic retinopathy(ETDRS) visual loss due to macular odema is reduced by 50% with immediate focal laser photocoagulation treatment.^6^ Initially, laser photocoagulation in PDR was performed by Argon blue-green laser introduced by L’Esperance in 1968, and krypton laser in 1972 followed by diode lasers, however due to longer treatment time and increased incidence of collateral damage and inflammation they are currently replaced by shorter pulse duration lasers.^6^,^7^ The longer pulse duration has been associated with more tissue damage and spread of energy to surrounding retinal layers.^8^

The advent of intravitreal anti VEGF agents has revolutionized the management of diabetic eye disease over the last decade. Mechanism of action of these agents is the inhibition of various forms of endogenous VEGF, however the duration of effect is short and intravitreal injections need to be repeated.^9^,^10^

Pattern scan laser (PASCAL) is a short pulse laser with a pulse duration of 20-30 millisecond, has been associated with much lesser risk of induced macular edema in comparison to argon laser which has pulse duration of 100 msec.^11^ Some authors claim, PRP alone with short pulse lasers can not only treat PDR but also significantly reduce the DME.^12^,^13^

Therefore, the rationale of conducting this study was to observe the effect of short pulse laser (PASCAL) PRP alone versus PRP augmented with intravitreal bevacizumab (IVB) injections in patients with PDR.

## METHODS

This was a randomized clinical trial carried out at Armed Forces Institute of Ophthalmology, Rawalpindi, Pakistan from Jan 2015 to Aug 2016. Sample size was calculated on the basis of Open EPI info calculator and appeared to be 60 eyes. Seventy six eyes of 50 patients were registered (38 in each group). Two eyes of a patient was considered separately. All those diabetic patients who were newly diagnosed as having PDR with DME and no history of previous treatment were included in the study. To cater for the confounding role of other variables, patients with poor diabetic control (HbA1C > 7.0%), hypertension (>140/90), significant lenticular changes, traction, advanced diabetic retinopathy, cystoid macular edema (CMO) and subretinal serous elevation were also excluded from the study. Written informed consent was taken and demographic details were noted.

Detailed ophthalmic clinical examination of all the registered patients was carried out that included uncorrected distant visual acuity (UCVA), best corrected visual acuity (BCVA) and slit lamp fundus examination. In order to document the central macular thickness (CMT) and rule out presence of cystoid macular edema, macular traction and serous elevation, Optical coherence tomography (OCT- Topcon 3D OCT-1 Maestro) was carried out. All the subjects were randomly assigned to two groups (Group GP and Group GI). PASCAL (Streamline 532 nm Pattern scanning laser) alone was performed in group GP while PRP along with intravitreal bevacizumab (IVB) in Group GI. BCVA was recorded and OCT was repeated at 03 months after starting the treatment.

### Procedure

Patients in both the groups (GP and GI) were treated with Pattern scanning laser (PASCAL) using Quadraspheric (VOLK) lens by second author under topical anesthesia (proparacaine hydrochloride 1%). Two thousand burns were applied at 1^st^ session with 20 msec pulse duration, 200um spot size and power ranging from 350-600 mW, titrating the burn intensity until mild grey reaction was achieved. PRP was repeated at 04 weeks and 08 weeks with an additional 800-1000 burns. Patients in group GI were subjected to IVB injection (2.5mg/0.1ml) one day after PRP session and repeated monthly for 03 months. All sessions of laser and IVB injections were performed by the second author.

### Statistical Analysis

Statistical package for social sciences (SPSS 22.0) for windows was used for statistical analysis of data. Mean and standard deviation was noted for continuous variables while frequency distribution for categorical variables was recorded. Paired sample t-test was used for analysis of pretreatment and post treatment BCVA as well as CMT within a group, while independent t- test (p < 0.05 significance level) was used to analyze induced change in BCVA and CMT between the two groups (GP and GI).

## RESULTS

Seventy six eyes of 50 patients (38 in each group) were treated with three sessions of PRP alone and PRP with IVB in Group GP and GI respectively. Out of total 76% (24) were males and 24%(6) were females in group Gp while 64% (21) males and 36% (10) comprised the Group GI. Out of total, 16 patients had bilateral involvement while there were 14 right and 20 left eyes. Mean age of the patient in group GP was 57.47±6.08 years while that in group GI was 55.69±6.58 (Table-I). Mean BCVA and Mean CMT before treatment at 03 months after starting the treatment in both the groups GP and GI is depicted in Table-I. The magnitude of induced change in BCVA was 0.09±0.15 in GP while 0.22±0.04 in GI groups while mean induced change in CMT after treatment was 77.44±90.30 and 117.50±93.82 um in group GP and GI respectively (Table-II).

**Table-I:** Mean values of various variables.

Variables	N	Group-GP	Group-GI
Age	38	55.69 ±6.58	57.47± 6.08
Pretreatment BCVA	38	0.65±0.42	0.64±0.39
Post treatment BCVA	38	0.55±0.34	0.42±0.35
Pretreatment CMT	38	391.69±163.46	429.17±178.24
Post treatment CMT	38	314.25±85.70	311.67±96.34

**Table-II:** Induced change in visual acuity after treatment.

Groups	Induced change in Central Macular thickness (CMT)	Induced change in Best corrected visual acuity (BCVA)	P value
GP	77.44±90.30	0.09±0.15	P < 0.001
GI	117.50±93.82	0.22±0.04	P < 0.001

## DISCUSSION

PDR and DME are two manifestations of Diabetic eye disease that are responsible for visual loss in majority of the patients and they are treated primarily with panretinal photocoagulation and intravitreal anti VEGF respectively. However, recent use of anti VEGF agents in PDR has also shown promising results not inferior to PRP.^9^,^14^ The proposed mechanism of IVB in resolution of both DME and PDR is binding to all forms of endogenous VEGF which is responsible for pathogenesis of DME as well as PDR.^15^,^16^ Avery et al also concluded the antiangiogenic as well as anti exudative role of IVB in their study.^17^ On the other hand laser photocoagulation has been used in the treatment of PDR for the last couple of decades and the proposed mechanism is decreasing ischemic drive, converting ischemic to anoxic retina and therefore, leading to regression of retinal new vessels. The recent use of Pattern scan laser (PASCAL) which has a short pulse duration has shown beneficial effect on DME when used for combined presentation of PDR with DME.^5^,^6^,^12^,^18^,^19^

We, in our study combined the two treatments in patients with simultaneous presentation of PDR and DME. In our study it was observed that there was significant improvement in BCVA and CMT in both the groups (Table-II). Mean change of BCVA at 03 months after treatment was 0.09±0.15 in group GP while mean change in CMT was 77.44±90.30um which was statistically significant. Similarly the mean change in BCVA and CMT in group GI was 0.22 ± 0.04 and 117.44 ± 93.80 um respectively (Table-II). This reduction in CMT and improvement in BCVA is also concluded by Gaucher et al in their study where they found out reduction in CMT after two sessions of PASCAL.^20^

In comparing the two groups (group GP and GI), the induced change in BCVA and CMT was more in group GI where combined treatment of PRP with intravitreal IVB was used (Table-II). Several other authors such as Kumar H et al and Simunovic MP et al have also found out superior visual outcome in patients when anti VEGF agents were used as adjuvant to PRP in PDR in their studies.^9^,^21^ We have found out better visual and anatomical outcome with adjuvant use of anti VEGF agents in PDR especially when simultaneously present with DME. Several other studies have concluded the beneficial effect of anti VEGF agents in DME and PDR.^22^-^24^

### Limitations of the study

We could not consider presence of other comordities such as hypertension, diabetic nephropathy and the duration of diabetes in each group. Moreover we measured short term post treatment effect of 3-4 months.

The results of our study clearly depicted the superior visual outcome when IVB is combined with PRP in treatment of PDR with DME and we think our findings are significant and worth reporting. However we recommend that studies with longer duration, recruiting larger cohort and considering all the cormordities and confounding variable should be consider to yield more comprehensive results.

It is concluded that IVB along with PRP is effective and has superior outcome to PRP alone in patients who have simultaneous presentation of PDR with DME.
